# What rare cancers have in common. The making of lists of (very) rare cancers and the coordination of medical work

**DOI:** 10.3389/fsoc.2023.1148639

**Published:** 2023-08-30

**Authors:** Héloïse Pillayre, Sylvain Besle

**Affiliations:** ^1^Centre Léon Bérard, Département SHS, Lyon, France; ^2^Université Claude Bernard Lyon 1, Lyon, France

**Keywords:** rare cancers, oncology, precision medicine, lists, coordination, rarity, boundary objects

## Abstract

This article aims to understand why medical actors recently published lists of rare and very rare cancers. It studies four lists of rare and very rare cancers based on interviews with the main actors on these lists and an analysis of medical articles in which these lists were published. It argues that these lists constitute boundary objects whose aim is to deal with the organizational challenges raised by precision medicine, which imply increasing the coordination work between various types of actors. Our work therefore allows a better understanding of the functioning of the recursive standardization process of a boundary object and, by analyzing how the category of rarity is built at the intersection of both professional and nosographic principles, shows the intertwining of the biomedical, organizational, and political aspects on which rests the practice of contemporary precision medicine.

## Introduction

Advances in precision medicine have produced an increasing subdivision of cancers, thus generating a disaggregation of the “cancer” object in favor of a multiplication of new medical entities now qualified as rare (Bourret, 2005; Castel et al., 2019), and the need for new classifications (Navon, 2019). Classifications are a classic issue in the epistemology of medicine, as they directly question the ontology of medical categories (Fagot-Largeault, 1989; Plutynski, 2018). Recent works have shown how precision medicine, also called “personalized medicine, renews the way of considering these classifications (Keating et al., 2016). These works show how genomics represent a turn to a “molecular gaze” in a Foucauldian sense (Rose and Miller, 1992; Rabinow and Rose, 2006; Rose, 2007; Navon, 2011). Some of the analyses also show how precision medicine, by producing a proliferation of new medical entities, engendered a need for “finer grained and more dynamic taxonomies” (Green et al., 2022),” and renews the way of considering rarity and of classifying tumors (Wadmann, 2023). However, the organizational dimensions of this proliferation of new medical entities in terms of the coordination of medical work, of the production and circulation of expertise, of the organization of—more and more individualized—healthcare pathways, and of the regulation of the production of new drugs has not been completely explored yet. In order to analyze how medical actors try dealing with the organizational challenges raised by the proliferation of new entities related to the emergence of precision medicine, this article analyzes the making of lists of rare and very rare cancers by these actors, since the beginning of the 2000s. The article argues that, facing this proliferation of new entities, medical actors feel the need to group the latter again following their incidence rate, thus building a “rare” category. It shows that the appropriation of rarity by medical actors in the field of oncology is therefore a response to the difficulties raised by the necessity of building more dynamic taxonomies, which questions the possibility of coordination between these actors.

These lists of rare and very rare cancer, which reference what there authors consider as rare entities, their coding in the International Classification of Diseases and their incidence or prevalence rate, do not renew existing nosological categories. They do not aim at drawing disease categories relevant for diagnosis. They aim to label certain types of cancers as rare, thus imposing rarity as a relevant medical criterion for identifying certain cancers and grouping them into a comparable category. The objective of this article is to explain why these lists have appeared and show the fundamental role they play in the coordination of medical work on new entities that are part of uncertain and dynamic categories: they aim to build a governable object (Lascoumes, 1996), to organize specific healthcare pathways, to facilitate the circulation of expertise and to coordinate action with the medicine agencies. This article shows that rarity does not constitute a medical category in itself, but both a nosographic and organizational category whose construction aims to coordinate different actors confronted to an increased complexity related to the multiplication of medical entities. It is inspired by different works that demonstrate how organizational, political and epistemic dimensions are deeply intertwined in the making of the categories that have emerged from precision medicine (Cambrosio et al., 2006; Green et al., 2022). They also show how these classifications constitute a material basis for creating and regulating the production of new medicines (Navon and Eyal, 2016), but also how they can create new types of identities for patients (Jutel and Nettleton, 2011) and potentially engender new types of inequalities and discriminations. By doing so, this article adds to this literature by showing how this collective appropriation of the notion of rarity at very different scales is a response of medical actors to the organizational aspects raised by the proliferation of new entities, which implies the production of categories that rest on an increased intertwining of biomedical, organizational and political dimensions.

The issue of rare diseases is, however, not new in the field of healthcare. This category of diseases was put on French and then European political agendas under the impetus of patient associations (Huyard, 2009a,b; Rabeharisoa et al., 2014) who joined together in a common European movement, Eurordis. The political work done by patients' associations contributed to put forward rarity as a relevant criterion for public health policy. This led to the establishement of a standardized European rarity threshold based on prevalence (1 person per 2,000), linked to the European regulation on orphan drugs. Caroline Huyard uses the concept of boundary object to characterize rare diseases and shows that the construction of this category of rarity derives both from the specific experience of the disease put forward by patients' associations and from the desire of the European Union to achieve an alignment of regulations with the European common market. But Huyard points out that at the time of her survey, the category of rarity had little resonance among health professionals.

However, the making of lists of rare diseases by health professionals challenges this idea, from the foundation of Orphanet, a database on rare diseases launched by a French geneticist in the 1970s, to the recent proliferation of lists of rare and very rare cancers. This article raises the question of the appropriation of the notion of rarity by medical actors. It shows how lists of rare cancers have been constituted as boundary objects in order to coordinate medical work. The notion of boundary object, initially developed by Star and Griesemer (1989) and used by Huyard, describes entities that are both abstract and material, around which different communities organize and structure themselves. This article shows that lists of rare cancers constitute boundary objects, in the way that they are a material object making reference to an abstract category, and which is used by different medical actors to coordinate with each other. The interpretive flexibility of the boundary object is a key element in this process, in the sense that it allows the object to be reappropriated by different communities according to various local issues, and therefore allows these communities to work together. This aspect has been already analyzed through the prism of the coordination of medical work by using a ≪ shared space ≫. But another dimension of the boundary-object remains quite unexplored, which is the recursive dimension of the boundary object: indeed, Susan Leigh Star explains how ≪ boundary objects are constantly caught up in a ≪ back and-forth between the ill-structured and wellstructured use of the arrangements ≫. The construction of a boundary object thus constantly oscillates between attempts at standardization, never completely achieved since the process of abstraction cannot capture the plurality of complexity of the multiple arrangements, and constant work to redefine this object to make it more tailored to local uses, thus working at new standardization attempts. Star shows that this constant restandardization movement is related to the fact that standards create ≪ residual categories ≫, which caracterize categories at the margins of standards and left apart by the standardization process (Star, 2010). Rare categories, because they are numerous, heterogeneous, and difficultly caught up in this standardization process, can be considered as residual categories.

This article focuses on the work done by medical actors concerned by these “residual categories” to understand how they build arrangements to allow themselves to achieve some sort of consensus on what is rare and what is not rare. The demarcation of rarity oscillates between, on the one hand, attempts at standardization, never completely stabilized, which emanate from different types of actors and that aim to establish epidemiological thresholds, and, on the other hand, more specific circumstances where the concept of rarity is mobilized and can push certain actors to free themselves from these standards and produce new definitions of rarity.

In order to better characterize the constant back-and-forth between wellstructured and ill-structured uses of the concept of rarity, aiming to trace how different medical actors effectuate a boundary work to conceptualize, circumscribe and define rarity, this article proposes the notion of jurisdiction (Abbott, 1988). Jurisdictions characterize, for Abbott, the link between a profession and its work: that is to say, the way in which some actors claim an expertise about specific areas or entities. This article will thus show how professionals use the notion of rarity to define, maintain or expand their domain of expertise over specific medical entities. As Abbott claims, “jurisdictional boundaries are perpetually in dispute, both in local practice and in national claims.” By understanding how different medical actors circumscribe their areas of expertise by defining rare entities, this article will thus show how the study of these jurisdictional struggles helps understand the recursive standardization of rarity as a boundary object.

This article is also inspired by more recent works that use or criticize Abbott's concept of jurisdiction. Firstly, this article is inspired by the analyses of Timmermans, who shows how “professions gain jurisdiction when they control their skills through abstract knowledge and technique” (Timmermans, 2002), how they attempt to sway legislation, and how this leads to a “politics of expertise,” since professionals seek to maintain the boundaries of their expertise by monopolizing abstract knowledge and technique. This will help us to understand how medical actors, by effectuating a boundary work around the notion of rarity, seek to create their own jurisdiction, characterized by rare entities. Secondly, this article is inspired by the work of Eyal, which criticizes Abbott's analysis by explaining that expertise should be understood with much more fluidity, showing how actors do not always seek to maintain a jurisdiction, aiming at keeping a monopoly of expertise, but sometimes at the opposite aim to make knowledge and expertise circulate (Eyal, 2013).

Drawing on these studies, this article will first analyze the creation of lists of rare cancers to understand why medical actors effectuate a boundary work around rarity, showing how they publish such lists to make these residual categories visible for public actors and to claim funding for their research, and to claim specific healthcare pathways for these patients. By so doing, they seek to create and maintain a jurisdiction on these residual categories, thus contributing to the standardization of the boundary object, which allows them to stabilize the contours of this jurisdiction.

Then, the article focuses on the making of lists of very rare cancers, residual categories left apart by the standardization of rarity. It shows how medical actors concerned by very rare cancers aim to coordinate biomedical work about them, and to communicate with actors from the regulation agencies about the specificities of these entities. This ill-structured work around a new boundary object aims at the contrary to make expertise circulate rather than monopolize control over a jurisdiction.

## Methods

This article is based on the study of four lists of rare cancers that were published between 2007 and 2020. It is part on a broader research program on the Europeanization of healthcare for rare cancers. The first list, Orphanet, is the first list of rare disease that has been made in Europe in the 1990's. This list, which includes a list of rare cancers, is important because it is a reference in Europe concerning rare diseases, and a basis for European policies on rare diseases.

The second one, RareCare, is the result from a research program on rare cancers funded in 2007 by the European Union. This list has been chosen because it is the most important list of rare cancers at the European level, used by both medical actors and European authorities to create reference networks for these cancers. The two other lists that have been chosen are specific to sarcomas and childhood cancers and reference ≪ ultra ≫ or ≪ very ≫ rare tumors and were published in medical journals in 2019 and 2021. They have been chosen because they are the only lists published in oncology journals that reference ultra-rare and very rare tumors. Indeed, both sarcomas and childhood cancers are two specific fields in oncology that deal mostly with rare entities, and medical actors from these areas feel the need to distinguish between rare and ultra rare entities. The analysis of these three lists makes it possible to consider rarity at different granularity levels, thus helping us understand the constant standardization and destandardization of the boundary object.

A study of six medical articles that got published around these lists has also been conducted. These articles have been chosen because they are the ones in which the lists have been published, or articles that give comments on these lists or use them. The study of these articles help specify both the reasons for the making of the lists and the way they have been made.

A difficulty in studying lists of rare and very rare cancers consists in the diversity of actors involved in their construction and their use, considering the fact that they aim to coordinate these actors. The article issued from the RareCare study is signed by 22 co-authors, mostly epidemiologists and people in charge of cancer registries in different European countries, but also oncologists and molecular biologists (Gatta et al., 2011). Three of them have been interviewed.

The article presenting the list of ultra-rare sarcomas is signed by 60 co-authors involving epidemiologists, clinicians, researchers and hospital directors from all over the western world (Stacchiotti et al., 2021). Eight of them have been interviewed. The article presenting the list of very rare childhood cancers is co-signed by 18 authors, oncopediatricians and epidemiologists (Ferrari et al., 2019), eight of whom have been interviewed. One epidemiologist, Annalisa Trama, is central, as we will see, in the making of these lists is present in these three articles.

Semi-directive interviews with these actors lasted between 1 and 2 h and were based on an interview guide which aimed to understand which place the actors occupied in the making of the lists, why they participated to this process and why they find these lists useful. Another part of the questions aimed to understand from which type of network these actors were part of and the link they have with the European institutions, and especially expert committees of the European Commission.

### From rare diseases to rare cancers

The first official classification of diseases dates to 1893 when a French physician, Jacques Bertillon, was commissioned by the International Statistical Institute to establish a classification of causes of death at a congress in Vienna in 1891 (Bowker, 1996). This classification was subsequently revised five times in 10 years until 1938. At its creation in 1945, the World Health Organization (WHO) was entrusted with the evolution and update of this classification. In 1948, the sixth revision became the “International Statistical Classification of Diseases, Injuries and Causes of Death” (ICD), which moved away from listing only causes of death to broaden its focus on morbidity in general. In 1967, the WHO stipulated that Member States should use the latest revision for their health statistics on morbidity and mortality. In addition, the classification of cancers was separated from the ICD in 1976. From this time, cancers have been classified in a separate list, the ICD-O (International Classification of Diseases for Oncology). This separation is explained by the need of oncologists to have lists both on the topography and morphology of tumors: that is, the location of cancer cells (breast, lung, uterus) and their form (carcinoma, mesothelioma, sarcoma).

To understand the emergence of lists of rare cancers, it is necessary to start with the history of lists of rare diseases, which emerged earlier. The history of the making of lists of rare diseases is linked to the realization of certain physicians, confronted with difficulties related to the diagnosis of certain diseases, of the shortcomings of the ICD, which they thought did not reference well enough the rarest diseases. Until this point, rare diseases thus constituted ≪ residual categories ≫, only referenced in classifications in categories such as ≪ Not elsewhere categorized ≫, or ≪ None of the above ≫, In order to better characterize these residual categories, a French geneticist and physician, Ségolène Aymé, who had also studied epidemiology and bioinformatics, was confronted in her clinical practice with patients having rare diseases that she did not hear about before and that were not referenced in the ICD. In the 1970s, she set up a database on rare diseases, initially for her own use. This database became Orphanet in 1997.^1^ This database does not only reference rare diseases better than what was the case in the ICD, it also compiles information on the epidemiological indicators of rare diseases via a systematic collection procedure in medical journals and registries.

Orphanet quickly took on a European institutional dimension. The European Commission started to use the list as a model to trace and codify rare diseases in the European Union. This process of recognizing rare diseases as a category asking for a specific public response has led to a standardization of rarity at the European level, under the aegis of the European Commission, based on a prevalence threshold, following an important mobilization of patient associations (Huyard, 2009a). The creation of this list thus characterizes the material process by which Ségolène Aymé created a boundary object, aiming to seek cooperation between scientific actors working on these residual categories and political actors within the European Commission. The creation of this database has also contributed to making the creator of Orphanet a central and unavoidable figure of the cause of rare diseases, present in most commissions at both the French and European levels. The making of this boundary object therefore allowed her to create her own jurisdiction over these residual categories, by establishing control over a new public problem and its government, hereby establishing herself as the main interlocutor of the European Commission concerning rare diseases. For example, Ségolène Aymé was appointed as president of the ≪ Rare Disease Task Force ≫ in 2004, the first committee of experts on rare diseases that was created by the European Commission. This appropriation of this public problem was only made possible by the construction of a boundary object that allowed her to translate a medical expertise into the political field.

Specific lists of rare cancers emerged later, in the mid 2000s, following the making of rare diseases as a boundary object between the medical and the political fields. Indeed, several actors from the oncology field wanted to stress out the specificities of rare cancers, which cumulate both the specificities of rare diseases and of cancers. Indeed, the idea that these rare cancers present common specific difficulties compared to rare diseases regarding care and management emerged in the mid-2000s. Some clinicians emphasized the difficulties common to the treatment of all rare cancers, which nevertheless group very heterogeneous entities: diagnostic difficulties, lack of standardized protocols, and the difficulties of patients to find people who share the same conditions (Raghavan, 2013). As this oncopediatrician, member of the ExPERT group emphasizes it, precision medicine was at the origin of this multiplication of rare tumors:

The recurrent molecular anomalies found in a certain number of patient groups are synonymous with a certain prognosis, a certain treatment and so on. What is very complicated is that at the time we had 3 groups of treatments, today we must have 15 because we are segmenting more and more. And so, diseases that were relatively frequent... when you have a disease that is frequent and you make 10 groups, it doesn't become 10 frequent diseases, it becomes 10 rare diseases. It makes things more complex.

This excerpt shows well how medical actors perceive the multiplication of rare entities engendered by the emergence of genomic medicine and the related complexity of managing care and research about them. This subdivision of disease categories into multiple subtypes has already been well shown (Bourret, 2005; Green et al., 2022), as well as the renewing of their relationship with diagnosis and of the characterization of illnesses (Navon, 2011).

But if the way in which precision medicine has renewed existing classifications has been well analyzed, it is not the case for the apparition of a ≪ rare ≫ category, which is not about subdividing existing entities, but about thinking of how to deal with this proliferation of new entities by defining, labeling and grouping them into new categories in order to organize expertise and care about them.

The first list of rare cancers was published within the framework of the RareCare project, funded by the European Union in 2007, bringing together oncologists, epidemiologists and geneticists from the main countries of Western Europe and which was the first European funding program specifically dedicated to rare cancers, mostly aiming at identifying and quantifying them. Thus emerged a progressive standardization process of a new category inside the standardized rare disease boundary object.

In order to build the first list of rare cancers, members of the RareCare project used the ICD-O-3 (3 means the third version) classification and identified rare cancers according to their incidence rate defined as <6 per 100,000 per year. From this list, different groups and subgroups were formed. The RareCare list also references the topography and morphology codes from the ICO-3 and the incidence rate of these cancers:

The recursive standardization of the object ≪ rare diseases ≫ did not stop, however, with the making of the ≪ rare cancers ≫ boundary object apart from the ≪ rare disease object ≫. More recently, a list of very rare cancers has been developed for childhood cancers. It has been published by a European network of pediatric oncologists, which groups together clinicians who struggle to treat these diseases. These oncopediatricians started to build national networks from 2000, in order to share expertise. In Poland, the Rare Pediatric Tumor Study Group (PPRSTG) was launched in 2002, in Germany the STEP (Seltene Tumoren in der Pädiatrie) in 2006, in France the FRaCTurE group (Groupe FRAnCais Des TUmeurs Rares de l'Enfant) in 2007. Projects financed by the European Commission have helped to structure these networks. In 2008, these networks grouped together and built the European ExPERT group, specialized in very rare childhood tumors. This list is based on the ICD-O-3, cross-referenced with data on incidence rates taken from the RareCare study. The making of this list has been, as for the RareCare list, accompanied by the standardization of the category by the definition of a threshold. Indeed, members of the ExPERT group have defined the threshold for very rare as an incidence rate of <2 in 1,000,000 per year. According to this list, 11% of pediatric cancers are very rare. These very heterogeneous tumors include both cancers that are common in adults but rare in children, and rare cancers that are specifically pediatric (hepatoblastoma, pleuropulmonary blastoma, pancreatoblastoma, etc.).

At the same time, another network of experts started to build another category of ≪ very rare tumors ≫, also identifying specific entities from the RareCare list. The Connective Tissue Oncology Society (CTOS) brings together sarcoma specialists from around the world, and has commissioned a panel of experts in 2019 to develop a list of ultra-rare sarcomas. The committee brings together specialists from Europe, North America, Asia and Australia, covering all disciplines involved in sarcoma research and care (epidemiology, pathology, molecular biology, surgery, radiotherapy, medical oncology). As with pediatric cancers, these experts took the ICD-O classification of soft tissue and bone tumors and cross-referenced it with epidemiological data from the RareCare studies to extract those classified as ultra rare. The CTOS-appointed expert committee set the threshold for ultra-rare at an incidence of <1 case per 1,000,000 per year. As we see, this threshold this resulted in 18% of sarcomas being considered as ultra-rare. The resulting list was updated as new versions of the ICD-O emerged.

This multiplication of lists of rare and then very rare diseases and cancers across time testimonies for an increased need of medical actors to better define, label, count and circumscribe these residual categories. This need gave raise to a recursive and progressive standardization of imbricated boundary objects at very different scales and for different usages: firstly rare diseases, then rare cancers, then very rare cancers. We thus have to wonder how and to what extent is the appropriation of the category of rarity the response to the organizational challenges raised by the proliferation of new entities by imposing a new criterion for grouping them.

### Why do medical actors publish lists of rare cancers?

Indeed, defining some cancers as rare not only means better referencing them as individual entities but also thinking about what they have in common that requires grouping them into a specific category, which is not anymore referenced as ≪ Not elsewhere categorized ≫ or ≪ None of the above ≫ anymore, but as ≪ Rare ≫. In other words, it means admitting that rarity is a relevant characterization for constructing a space of comparability between entities that would otherwise be completely heterogeneous.

Medical actors decided to characterize and to group these residual categories into a ≪ rare ≫ category for two reasons. The first one, epidemiological, aims at defining and counting rare cancers in order to create a boundary object that allows medical actors to represent the important number of these residual categories in order to build a significant public problem for which public authorities should find solutions. As such, grouping rare tumors is a strategy to make visible residual categories that were left apart by previous European politics on cancer: these lists deeply intertwine epistemological and political dimensions.

The second is based on the need to organize specific healthcare pathways for these rare entities to ensure that patients have access to clinical expertise that might be scarcer than for other cancers. But of course, the creation of a new boundary object which is ≪ rare cancers ≫ raises important power relationships between medical actors who aim to circumscribe and establish control over the new jurisdictions created by the emergence of these new categories.

#### Publishing rare cancers lists to build a governable object

The making of lists of rare cancers in the mid-2000s consisted for these medical actors in constructing a boundary object that allowed them to translate their difficulties to make them both perceptible and governable by public authorities. Epidemiological issues played an important role in the construction of such categories, since they made it possible to construct and circumscribe a rare category and to evaluate its importance in numerical terms. This epidemiological work is thus directly in line with what Vololona Rabeharisoa and her colleagues have described as a logic of numbers and a logic of singularization (Rabeharisoa et al., 2014). Indeed, the latter emphasizes not only the heterogeneity of entities, but also what these entities have in common by aggregating them in order to release a logic of number and make it worthy of attention.

Researchers participating in the European RareCare study, having circumscribed the category of rare cancers, have shown that they represent about 20% of all cancers (Gatta et al., 2011). The title of the article is “Rare cancers are not so rare.” By this oxymoron, the authors imply that, by a logic of aggregation, rare cancers taken in isolation are no longer rare if the whole category is considered. The construction of this category is thus part of a logic of numbers that might seem paradoxical at first sight. This is what the epidemiologist in charge of the RareCare program financed by the European Union, and the main actor in charge of the RareCare list, mentioned in an interview:

So, I'm an epidemiologist and basically since the last eleven years I've been studying mainly rare cancers because we initiated a European project during which we proposed a definition and a list of rare cancers. In the framework of this research I started of course to get in contact with most experienced oncologists, pathologists, radiologists, you know, surgeons of rare cancers and once we defined this list, we also decided that it was important to have data showing that rare cancers, because they are rare, so their frequency is low, are better treated in expert centers. So, we basically used data to provide evidence that rare cancers are not so rare. That together they're a lot. Calling for, you know, for priority, to give priority to rare cancers at national and European levels.

This grouping work allows the authors of the RareCare study to stress the numerical importance of the category of rare cancer, thus calling for specific European politics specifically directed toward them. The emphasis on the numerical importance of this category then contributed to put the issue of rare cancers on the European agenda and also to highlight the issue of quality of care for this type of disease. In this sense, rarity is not a purely medical concept, but the very idea of grouping residual categories into this category is in itself political: rare cancers are grouped to make them worthy of political attention. The construction of the “rare cancer” boundary object, based on a standardization process of epidemiological thresholds, then makes it possible to call for a specific treatment in terms of organization of care. As the same epidemiologist explains:

So, basically with the first project we identified the list of rare cancers, we showed there were differences in survival across member states. And we started reasoning about possible reasons for differences we thought that they had to do with the different healthcare organizations, which would imply the type of quality of care, because…we had to understand to what extent rare cancers patients are referred to the appropriate centers of expertise. So, we thought that one of the possible reasons for these differences was also the different quality of care provided in the member states.

The actors involved in RareCare, by constructing lists of rare cancers, thus constitute the material support of a boundary object and make the latter of attention for the European Commission, which no longer has to deal with a multiplicity of heterogeneous entities that are more complex to manage. The production of this boundary object also allows them to construct a governable public problem by highlighting its numerical importance and to call for a specific treatment. The translation of these issues into the political field, allowed by the making of a boundary object, is facilitated by the fact that some actors from the medical field have been recruited in expert groups of the European Commission in order to implement a specific organization of care. For example, Annalisa Trama has been attributed a place in the EUCERD (European Committee of Experts on Rare Diseases), the expert group which replaced the Rare Disease Task Force in 2009. In this expert group, Annalisa Trama represented the community of rare cancers and advocated for putting specific healthcare pathways for rare cancers into place, alongside with Ségolène Aymé.

#### Publishing rare cancers lists to better organize healthcare pathways

Some studies have shown how the renewing of classifications related to genomic medicine did not only have epistemological implications, but also political and organizational ones (Green et al., 2022). In this way, rare cancers are a response to the organizational challenges raised by the profiferation of new medical entities engendered by the emergence of precision medicine: if medical actors group rare cancers into a category to seek political attention, it is in order to claim a specific and common organization for rare cancers.

Since the beginning of the 2000s, under the impetus of patients associations, the treatment of rare diseases has tend to be increasingly centralized in expert hospitals that are concentrating cases around a specific disease. This organization is the result of several national plans for rare diseases in various European countries. Initiatives have also been taken to set up this type of organization at the European level, by creating European rare disease networks (ERN) organized around centers of reference. Four of these networks are dedicated to rare cancers: pediatric cancers are covered by the PaedCan network, sarcomas by the EuraCan network dedicated to rare solid cancers, the EuroBloodNet network is dedicated to hematological cancers and the Genturis ERN to tumors with genetic risks. The delimitation of the scope of action of these networks derives directly from the construction of lists of rare cancers.

Both medical and political actors have to deal with the tension between the heterogeneity of residual categories and the standardization process required by the construction of the rare cancers' boundary object. Indeed, each cancer cannot have a completely individualized care pathway so it implies to group them. Lists have to become classifications that can aggregate residual categories into groups, a need expressed here by a French physician, specialized in sarcomas:

Now the challenge will also be to use these classifications and to know how to group things together. Because [...] what I'm saying seems to be aberrant when I've just said that it's important to dismantle, but it's also good to know how to reorganize groupings because we don't necessarily have 150 therapeutic strategies and so there are also subtypes that can cross from a diagnostic point of view and from a treatment point of view. And so today the effort to be made is that we have seen what is different, we must also see what is common. Identifying common vulnerabilities for therapeutic strategies that can be identified for different subtypes is important.

This excerpt shows the organizational challenges that have been raised by the emergence of precision medicine and the subdivision of tumors into multiple rare entities, which requires grouping heterogeneous entities into different “categories” to be able to build specific healthcare pathways. However, this process of grouping raises tensions between political and medical actors. The way of grouping rare cancers in order to build the European reference networks has raised a debate between the members of the European Commission, who wanted to limit the number of networks in order to facilitate their management, and the actors of RareCare, who wished a greater of networks were created as explained by Annalisa Trama:

And there was another output of Rarecare, we gave a list of twelve groups of rare cancers. Because we said that rare cancers are approximately 2000 different types of tumors but this is really difficult to perceive…so because for us it was really key to address the big families of tumors for which are specific referral pathways…which basically implies referent centers where expertise would be needed. We basically grouped these 2000 types of rare cancers in 12 families, which basically includes all childhood cancers, because they are all rare, and there is an ERN for childhood cancers, Paedcan. And there was an ERN dedicated to the rare hematological tumors, EuroBloodNet. Our original idea was to have other ten ERNs for what we call rare adult solid cancers. But the European Commission was against the fragmentation of cancers, as well as for rare diseases, so they asked the rare cancers and the rare diseases to try to combine the efforts. And so we ended up developing one ERN for the ten families of rare adult solid cancers which is Euracan. So, basically from the epidemiological data one of the big outputs for me to get involved in designing a bit the ERN was because of this concept of families that we discussed together.

The actors of RareCare finally decided to organize the grouping at two levels. Firstly, they created categories which constitute relevant entities for patient care pathways organized around expert hospitals. Next, these categories have themselves been grouped into twelve families of rare cancers (e.g., nervous system cancers, digestive system cancers, sarcomas, pediatric cancers). These tensions around the grouping of heterogeneous rare entities reveals the tensions of rarity as a boundary object between political and medical actors.

This process of grouping has quickly led to a rethinking of the notion of rarity, which no longer applies only to clinical entities but also to families of rare cancers. Indeed, epidemiologists and clinicians no longer consider only clinical entities, but which groups of entities are rare. Indeed, several rare cancers can be grouped together in a family that is not rare from an epidemiological point of view:

There is a big difference between a rare “family” of cancers and a rare cancer “entity” belonging to a common family of tumours. For example, metaplastic cancers of the breast are a rare cancer entity, with the same incidence as, say, pleomorphic liposarcoma. However, while it may well be equally problematic to do any clinical research exclusively focusing on both, the expertise needed to approach appropriately a metaplastic breast cancer will be relatively easy to find in the community. This does not apply to pleomorphic liposarcoma, for which referral centres, or networks, will inevitably be more difficult to find in the community.^2^

These tensions around the making of lists of rare cancers between medical actors and the European Commission have led to the transformation of the boundary object from a list into a classification. This transformation shows how the organizational questions raised by the emergence of rarity as a specific category around which organizing healthcare pathways led to reconsidering rarity as a whole, which is here not only defined by the establishment of an epidemiological threshold but also by a lack of ≪ expertise ≫, that is to say of knowledge about how to treat these tumors. This evolution in the way of considering rarity, embodied into the materiality of a list, which is getting transformed by the usages that different actors make of it, is typical of the plasticity of the boundary object. It also shows the deep intertwining of both epistemological and organizational dimensions in the grouping of these rare tumors.

#### A conflict of jurisdiction between rare cancers experts and rare diseases experts

Of course, as for every categorization process, the emergence of rarity as a boundary object raised important power plays, characterized by the willingness of certain actors to establish control over this new jurisdiction. The medical actors who tried establishing a jurisdiction over rare cancers ended up being confronted with Ségolène Aymé, who, as we have seen, was also trying to establish a jurisdiction over rare diseases, and thus about rare cancers. As Ségolène Aymé explains, she had troubles communicating with these new actors in the field of rare diseases, who were trying to assert a jurisdiction on a field that she perceived as her own:

So rare cancers are rare diseases... All childhood cancers are rare... And the cancer community has had a hard time accepting to join the rare disease community. I was welcomed like a cat amongst the pigeons. It's not easy... Well, that's normal, all communities have their culture. While finally they are in exactly the same type of galleys… they continue not to want to consider themselves completely on the side of rare diseases... and yet, Annalisa Trama, all that, we made efforts to get them into the Eucerd, into the working groups of the European Commission, but they continue to want to play their game... while basically, for orphan drugs, they are in the same boat.

These conflicts of jurisdiction that take place within the European expert groups of rare diseases are also reflected in discussions around the making of lists of cancers. Indeed, the list created by Ségolène Aymé, Orphanet, also references rare cancers. But if Orphanet contains, as a list of rare diseases, a list of rare cancers, they are not referenced in the same way as in RareCare. Indeed, Orphanet's list uses the standardized definition of rarity accepted at the European level in 1999, according to the incidence rate, while the RareCare study qualifies rarity based on the incidence rate as explain in this article:

We used a new incidence-based criterion for defining rare cancers. In Europe rare cancers are often defined according to the prevalence criterion of <50/100,000, in the same way as rare diseases in general. However, prevalence has shortcomings as a measure of cancer rarity since some cancers with low incidence but good survival will fall into the common category as good survival pushes up prevalence; examples are squamous cell carcinoma of the uterine cervix and thyroid carcinoma. Similarly, some commonly-occurring diseases for which survival is poor are considered rare because poor survival pushes prevalence down. Examples are adenocarcinoma of stomach and lung and squamous cell carcinoma of lung. These considerations suggest that incidence is better for defining rare cancers, and is also in harmony with the sub-acute clinical course of most rare cancers; whereas most rare non-neoplastic diseases have a chronic course so prevalence is a better measure.^3^

Researchers argue that this definition corresponds better to remission phenomena. Basing rarity on the prevalence threshold has resulted in the bad prognosis cancers being removed from the so-called rare cancers since their prevalence rate is higher. This definition was rapidly adopted, particularly by European epidemiological studies. It has also been adopted beyond Europe in various studies in Asia and the United States (Tamaki et al., 2014; DeSantis, 2017; Matsuda et al., 2019). This imposition of a new definition of rarity that fits more the specificities of rare cancers is thus representative of the recursive standardization process that affects boundary objects (Star, 2010) and the constant reappropriation by local actors who build other objects more tailored for their own use. This gave rise to different definitions of rarity and various establishments of epidemiological thresholds depending on the scope of the boundary objects. What is interesting to consider is that this multiplication of definitions of rarity is related to the willingness of certain medical actors to impose a jurisdiction on a field, thus extending or at the opposite restricting these definitions to more or less specific uses. This encourages to consider the important power plays that are at stake in this progressive emergence of rarity as a specific category in response to the organizational challenges raised by precision medicine.

### Very rare cancers: a new boundary object in construction

We have analyzed why medical actors decided to make lists of rare cancers, in order to build visible and governable objects for the European Commission and to implement specific healthcare pathways for these diseases. This work of grouping medical entities lead actors to question the very notion of rarity, going beyond the setting of an epidemiological threshold.

More recent initiatives aim to establish lists of very rare cancers. The study of the emergence of this new category shows that the proliferation of medical entities related to precision medicine implies for the medical actors involved to group rare entities into categories at different scales, These processes concern in particular rare oncology fields that are particularly confronted with a lot of rare tumors, whether they are not that rare (“common rare,” as medical actors sometimes say) or “very” rare. Here, on an even finer scale, an intertwining of nosographic and organizational dimensions is at play in the making of these new categories that aim at coordinating actors of biomedical innovation and political actors. However, contrary to rare cancers, the category of very rare cancers is not standardized at the European scale yet. This new boundary object is just starting getting used by medical actors for quite different reasons than the rare cancers' category, and reveals different types of coordination between the actors at stake.

#### Lists of very rare cancers: a new boundary object aiming at the circulation of expertise

At the beginning of the 2000's, some national and then international networks, among which the ExPERT group on very rare pediatric tumors, started to get interested in what they began to call ≪ very rare tumors ≫ as a specific category. The interest of medical actors in these residual categories raised with the realization among certain physicians-researchers that extreme rarity raises specific difficulties that are different from rarity. The creation of lists of very rare cancers is thus characteristic of the recursive back-and forth of the boundary object between wellstructured and ill-structured uses of the concept. Indeed, by standardizing rarity by imposing rarity threshold, the actors we studied before left again apart residual categories, very rare tumors, which are obviously part of rare tumors but also raise specific difficulties for clinicians. For example, very rare cancers may be confused by specialists with non-cancerous diseases, even in an expert hospital, as explained by a German pediatric oncologist specialized in very rare childhood tumors:

It's more difficult because these cancers are often primarily misdiagnosed for other types of cancers, or even other diseases, because with certain symptoms in certain age groups, you don't necessarily think about cancer diagnosis. So, for example, lung carcinoma in children is something which is very rare. And if a child presents with cough, and breathlessness and hemoptysis, or something like that, you don't necessarily think about the tumor first, but rather think about infection or something like that.≫

These difficulties were not raised by clinicians who dealt with “common” rare tumors. The boundary object “rare cancer” was then not able to completely cover the specificities of these entities and to respond to the specific organizational challenges that they raised in terms of coordination of expertise and care. Very rare entities are therefore characterized by the uncertainty that surrounds them clinically. These are residual categories that clinicians face difficulties to identify and for which there are no clear treatments guidelines, as explains a French specialist of sarcomas:

I became interested in this pathology for several reasons. The first reason is the clinical situations I was confronted with. So, it was a semester that was particularly striking for me considering the situations I saw, with many young adults, people who were my age, I was 25 at the time. They were teenagers, young adults with cancer who had clearly had major difficulties in terms of diagnosis. That's what struck me, that there were diagnostic errors that lasted for some time, and even when the patients were taken in charge in a reference center, the diagnosis was not always that simple. And I was quite struck by the variety of subtypes of sarcoma, and on the contrary by the fact that they were all treated in the same way, which seemed to me to be completely appalling, since there was a variety of pathologies that were clearly very different from a pathological point of view, from a biological point of view, from a molecular point of view, and yet we had very few drugs and always the same drugs that were used in these young people.

The notion of extreme rarity is rooted in the experience of clinicians who had specific difficulties treating some patients because of a lack of expertise. These difficulties encouraged them to reflect on how to make the available expertise better circulate and to create specific scientific networks, such as the ExPERT group, in order to improve the coordination of medical actors from different European countries. By doing so, these new networks created a new boundary object, “very rare cancers,” to characterize these residual categories.

The fact that extreme rarity is linked to a lack of expertise on specific medical entities characterized by certain mutations influenced the way these new networks of experts took it into consideration. What is interesting to consider is the fact that extreme rarity is not defined by these networks in terms of epidemiological thresholds, but by the fact that no network has already a specific expertise on most of these tumors. For example, in this excerpt of an article in which the ExPERT group published a list of very rare childhood tumors, is explicitly mentioned the lack of expertise as a specific criterion to characterize very rare tumors:

This means that all types of cancer occurring in childhood are rare: so how do pediatric oncologists define ‘rare tumors?' Rather than by their low incidence, rare pediatric tumors are generally identified by the fact that they are ‘orphan diseases,' in the sense that most pediatricians might encounter them only once in their working lives, there are few or no published reports on clinical experiences, it is difficult to establish shared treatment guidelines (and there are no evidence- based therapeutic recommendations available), and few or no cooperative groups have dedicated and structured projects, and financial support for studies on these tumors.^4^

Both the idea that there are no guidelines for these tumors and that no research group is interested in them is important to define extreme rarity. The previous excerpt therefore defines extreme rarity by the absence of medical actors who have developed a specific expertise on these tumors. In this situation of uncertainty about the very identity of the clinical entities at stake and about therapeutic choices, medical actors have therefore developed their own lists in order to identify entities that suffer the most from a lack of expertise. This shows how the very rare cancers' category is not a very stabilized boundary object yet, but is still in construction and is the object of negotiation between medical actors concerned with these residual categories. This constant and recursive work aiming to group medical entities into different categories of rarity shows the difficulty of medical actors to restructure the expertise and its circulation to adapt to the specificities of the new medical entities produced by precision medicine.

#### Publishing very rare cancers lists to coordinate biomedical work

Lists of very rare tumors then constitute a tool to organize the division of medical research and the production of expertise, in a context where the entities studied are particularly uncertain and the expertise concerning these cancers is disseminated in different research teams located in different countries, as explained in the following interview excerpt with an oncopediatrician who is part of the ExPERT group and who is a co-author of the article in which the list of very rare childhood cancers is presented:

Me: And what's the point of having such lists of very rare cancers?

It's useful not to get angry with your friends... The difficulty in our job is to manage to make projects while remaining diplomatic. Well, it's true that it allows us to be sure that we're actually within the frameworks, that we're not making mistakes, to be sure that... it's true, so I'm saying that, but it's true that in order to be sure that we're not encroaching on other people's groups by saying: we've decided to take care of that. So that's it. But it allows us to say to ourselves, ok, it seems logical to take care of it, and then to identify, above all if there are diseases that we would not have identified and that deserve it.

Contrary to what has been observed for rare tumors for which jurisdiction struggles were very strong between rare cancers experts and rare diseases experts, here it is more a question of cooperation and coordination between medical actors. Lists of very rare childhood cancer are therefore a means of controlling the heterogeneity of entities and of coordinating research work about them within different and heterogeneous medical actors. Lists are therefore made as boundary objects aiming to coordinate research work between specialists in order to distribute the production of knowledge as well as possible, by identifying entities for which there is no available expertise. In this sense, the aim of these actors is not so much about controlling a jurisdiction than about allowing the circulation of expertise among different actors and group of actors. As expressed by another French pediatric oncologist who is also a member of the ExPERT group and a co-author of the article where the list is presented:

It is absolutely essential to have this information in order to know who will take care of it. If you don't have this definition, I, who deal with rare tumors, who is part of... who is the president of the rare tumors committee, I will tell you that I will deal with hepatoblastomas, I will deal with sarcomas, these are rare diseases. These are very rare diseases and they can correspond to the definition of very rare diseases because we have been... we have worked in particular with the Italians, we have said that less than 2 cases per million inhabitants per year is a rare cancer. And so hepatoblastomas fit into that definition, retinoblastomas fit into that definition, most sarcomas fit into that definition. And so if I take this definition, I will be able to take care of all that. Except that I'm not a specialist and I'm going to be bad at it, so it's no use. There is a sarcoma group, there is a sarcoma group that will take care of these diseases and these sarcomas very well, and so if... it is important to identify which group will take care of them so that we don't step on each other's toes and so that people are actually specialized and work on their specialty, and get better little by little. So, it's really essential, if you don't define things in advance, first of all it's going to create tensions, tensions, completely sterile competition, and then you're going to dilute things and have people who will take care of everything and nothing and that's not going to advance, that's not going to help patients.

The making of lists of very rare cancers thus constitute a boundary object for new scientific networks to create an agreement on what are the specific entities at stake and to make the expertise circulate, and then to make sure that every one of these entities is part of the area of expertise of at least one research team. This aim to distribute well expertise is favored by the strategies of certain medical actors who have chosen these cancers for strategic reasons. Some physicians involved in these communities have also seeked for a niche encompassing themes where competition is less important, as expressed here by a German pediatric oncologist in the ExPERT group:

I think that most of the pediatric oncologists have first focused on the more frequent tumors because they saw more hum... more sustainable effect by improving the therapies for very... rare... hum for frequent tumors. And after, for most tumors, their concepts have improved a lot and the prognosis have improved, they... everybody has been looking for those where... who have been problematic. And so... in the first years of pediatric oncology, rare tumors have just been forgotten. They happened. But hum... but they were just treated, and nobody was caring. The other thing is... and this is more a hum... misanthropic interpretation, is that people like me were searching for their ecologic-... hum ecological niche, where they could find something, where they could do research without other people disturbing.

(Laughs) And to be honest for me it has been quite of both. And it's also been very pleasant to work in a niche where not one thousand pediatric oncologists wanted to work too.

Lists of very rare cancers thus constitute a boundary object aiming to improve the internal organization of international medical networks. What is more, this boundary object is useful for these networks to distribute a scarce expertise in the best way. In this sense, the creation of the category of “very rare tumors” consists in imposing a new way of managing the activity of medical actors that aim to take the best advantage of the competition between the latter.

#### Publishing very rare cancers lists to coordinate the relations with medicine agencies

Lists of very rare cancers can therefore be considered as a boundary object in the way that they constitute a common basis for discussion between medical actors who do not belong to the same field. But these lists also constitute a boundary object that aims to coordinate medical and political actors. Indeed, lists of very rare cancers are also intended to provide a basis for discussion with drug regulatory agencies. Concerning very rare cancers, there is very often no drugs authorized by national and European regulation agencies for these diseases. Drugs are therefore often used by clinicians off-label since randomized clinical trials are almost impossible to conduct because there are not enough cases. According to all the physicians-researchers interviewed, this is a clear dividing line between rare and very rare tumors, which is mentioned in the article that presents the list of very rare childhood cancer:

In fact, although all childhood cancers are rare, designing randomised controlled clinical trials is feasible for most paediatric tumours thanks to the well-established international cooperative networks, but it is unrealistic for many of the very rare paediatric tumours (it would take years to conclude a clinical trial).^5^

Lists of very rare cancers therefore make it possible to identify diseases for which it would be relevant to regulate the use of drugs in a context where randomized clinical trials are not feasible. They therefore serve as a boundary object between medical actors and the regulatory agencies at the European level with the aim of imagining a common and specific mode of regulation for the very rare clinical entities that are identified in the list and thus optimizing the production of new drugs for them.

Currently, there is no mechanism for bidirectional communication between clinicians, researchers, and regulatory bodies. We suggest that this could be achieved through regular mutual updates between the ultra-rare disease communities and regulators. In ultra-rare sarcomas, large studies are only possible with either long study durations and/or the involvement of a very large number of study sites (with corresponding quality-control issues).^6^

Aiming at producing new drugs, and with a logic of equity between all patients, whether they have very rare, rare or non-rare cancers, medical actors are using these lists as boundary object to call for new regulations to address the uncertainty that surrounds the conduct of clinical trials and the production of new drugs for very rare cancers:

In the area of ultra-rare sarcomas, disease-based discussions with regulatory agencies need to be planned on a regular basis, before embarking on the assessment of specific agents, including the incorporation of expert scientific advice, which affects the type of study protocol proposed for development. If an internal control arm is not feasible, optimizing the collection of external high-quality data by clinical registries should be encouraged. In the European Union, an opportunity not to miss is the involvement of the European reference networks: i.e., networks of cancer centers appointed by their governments to treat and research rare cancers. When label extension is not feasible, centralizing the use of selected off-label agents in sarcoma networks would be a way to guarantee appropriateness (…) while a higher degree of uncertainty should be tolerated; shared clinical decision making should be resorted to in order to manage such uncertainty.^7^

The creation of lists of ultra-rare cancers obeys logics that are different from those that animated lists of rare cancers. Very rare cancers constitute a boundary object created by medical actors that aim, internally, to divide up the work and the production of knowledge on heterogeneous clinical entities characterized by particularly significant clinical uncertainty. These lists also ambition to circumscribe the clinical entities for which it would be relevant to organize the regulation of drug production outside the gold standard of the randomized clinical trial. In this sense, the construction of “very rare entities” as a new boundary object is also a way, although at a different scale, to try to deal with the difficulties raised by the proliferation of new medical entities in terms of the production of new drugs. Indeed, this category might be useful to medical actors to question the traditional structures of expertise and care related to evidence-based medicine.

## Conclusion

The notion of rarity is taking an increasingly important place in the field of oncology due to the development of genomic technologies and precision medicine which tends to subdivide types of cancer into more and more entities (Bourret, 2005). This work shows the consequences of the development of precision medicine on the reappropriation of rarity by medical actors, and how the notion of rarity, born in the field of rare diseases under the impetus of patient's associations (Huyard, 2009a,b), has been reappropriated and adapted to the specific field of oncology.

To understand the reasons and consequences of this reappropriation, this article has focused on the lists of rare cancers, which constitute material objects aiming to circumscribe rare tumoral entities. It has shown that these lists constitute boundary objects that have been drawn up by health professionals, with the aim of coordinating medical work and responding to the specific challenges raised by these diseases: structuring networks of experts, identifying lack of knowledge, and giving access to new treatments. However, since the notion of rarity has been reappropriated by different medical and political communities, working on very different scales, these communities constantly produce new definitions of rarity. The notion of rare cancer is thus caught up, like every boundary object, in a recursive movement of standardization—establishment of thresholds from which a cancer can be considered rare—and adaptation to specific subfields of activity—sarcomas, pediatric oncology—which engender a multiplicity of ways of defining rarity. This recursive tension generates difficulties between the different medical networks at stake, which produce specific definitions of rarity and standards. In the end, far from being fixed, the notion of rarity is constantly evolving in the face of transformations in cancer care as shown by the case of ultra-rare cancers.

This study allowed us to better understand this recursive tension by characterizing the relationship between the different residual communities that are caught into it as a conflict of jurisdiction. This concept helped us to show that these lists allow medical actors to both circumscribe and extend their jurisdiction, in order to coordinate medical research activity, to build a visible and governable object, to organize care pathways and the relationship with the regulation agencies.

This article thus allows to understand how rarity, after having been claimed as a new identity basis by patients' organizations in the 1990s (Huyard, 2009a,b; Rabeharisoa et al., 2014) for a diversity of syndroms, has now been constituted as a relevant medical and organizational category aiming to coordinate various medical actors in oncology, as a response to the proliferation of new entities engendered by the emergence of precision medicine. Previous works on precision medicine have shown how the challenges raised by new diagnostic tools engendered a constant need for the revision of classifications (Green et al., 2022) as well as a constant recursive work between diagnosis and classifications (Navon and Eyal, 2016). They have also analyzed how precision medicine gave rise to an increased intertwining between organizational, political in the making of these categories (Wadmann, 2023). Instead of focusing on the constant revision of disease categories by the addition of new subdivisions (Bourret, 2005), this article analyzes how medical actors find a way to deal with the organizational challenges raised by the proliferation of new entities by appropriating the category of rarity. It shows not only how rarity is built on epidemiological thresholds, but is constituted as a performative organizational tool, which justifies to put specific regulations into place and to organize healthcare pathways for these particular types of diseases. By showing how this appropriation of rarity as a new category in oncology lies at the intersection of both professional and nosographic principles, this article shows how precision medicine requires the production of new categories, which rest on an increased intertwining of biomedical, organizational and political aspects, thus requiring analyzing them as “boundary objects” in order to better understand their plasticity and characterize these new entanglements. Further analysis should wonder what are the implications of this multiplication of rare medical entities, which at some point will make rare conditions a common thing, by analyzing its political implications, such as the questioning of the traditional structures of evidence-based medicine, and showing how it can create new boundaries between patients, new identities, and potentially new inequalities.

## Data availability statement

The raw data supporting the conclusions of this article will be made available by the authors, without undue reservation.

## Ethics statement

Ethical review and approval was not required for the study on human participants in accordance with the local legislation and institutional requirements. The patients/participants provided their written informed consent to participate in this study. Written informed consent was obtained from the individual(s) for the publication of any potentially identifiable images or data included in this article.

## Author contributions

HP: problematization, fieldwork, and writing. SB: coordination, editing, and reviewing. Both authors contributed to the article and approved the submitted version.

## Footnotes

^1^On Orphanet, see the work of Dagiral and Peerbaye (2012, 2013, 2016).

^2^Casali and Trama (2020).

^3^Gatta et al. (2011).

^4^Ferrari et al. (2017).

^5^Ferrari et al. (2019).

^6^Stacchiotti et al. (2021).

^7^*Ibid*.
